# Postmenopausal Uterine Leiomyomas and Chronic Lymphadenopathy: Exploring Epigenetic Changes and Pathophysiology

**DOI:** 10.7759/cureus.18274

**Published:** 2021-09-25

**Authors:** Leah O Grcevich, Alexis O'Connell, Maxwell J Jabaay, Jonathan T Scott

**Affiliations:** 1 Department of Research, Alabama College of Osteopathic Medicine, Dothan, USA; 2 Obstetrics and Gynecology, Women's Medical Center, Dothan, USA

**Keywords:** gynecologic oncology surgery, anemia, hmga2, menopause, abdominal mass, med12, abdominal lymphadenopathy, gynecologic surgery, uterine leiomyoma, uterine fibroid

## Abstract

Uterine leiomyomas (LM) are tumors arising from the non-neoplastic proliferation of smooth muscle cells within the myometrium. Like benign tumors, LM are not generally spread through the lymphatic system, and therefore should not be associated with lymphadenopathy. Herein, we present a case of a 60-year-old female who presented to the clinic with postmenopausal bleeding in the setting of sonographically evident uterine LM and abdominal lymphadenopathy. A lymph node biopsy revealed plasma cells and an eosinophilic material presumptively diagnosed as amyloid. She then underwent an abdominal hysterectomy for definitive treatment of LM. Surgical pathology confirmed the clinical diagnosis of uterine and cervical leiomyoma. Current literature suggests that genetic and epigenetic abnormalities contribute to the pathogenesis of LM in addition to hormonal signals such as estrogen and progesterone. It is unusual for LM to occur in post-menopausal women due to reduced hormonal influence. Therefore, this case explored an alternative mechanism of tumor proliferation. This case hypothesizes that genetic mutations and epigenetic changes resulting from chronic inflammatory offenses contributed to LM growth and lymphadenopathy.

## Introduction

Uterine leiomyomas (LM), colloquially termed uterine fibroids, are the most common benign gynecologic tumors [1]. LM arises from the non-neoplastic proliferation of smooth muscle cells within the myometrium [1]. By age 50, 70% of Caucasian women and 80% of African American women will have sonographic evidence of fibroids [2]. Several epidemiologic characteristics such as nulliparity, early menarche, age of last birth, higher education, nonsmokers, and intrauterine devices or oral contraceptive pills tend to have a greater risk of fibroids. Still, in general, the post-menopausal state is protective [3]. While there is a strong correlation between ovarian hormone levels and LM development, recent genomic studies of these tumors have identified novel pathways suggesting an inflammatory component of disease progression [4]. 

For example, *MED12* gene mutations have been implicated in the pathophysiology of multiple tumors affecting both sexes, including prostate cancer, and comprise one of the driving mutations involved in myocyte stem cell differentiation into leiomyoma [5]. Following transformation, plasma cells and extracellular matrix material interface with transformed myocytes leading to proliferation [4]. Estrogen and progesterone receptor activation are involved in tumor growth in part by activating cytokines and growth factors and inhibiting *p53* expression leading to dysregulation of cell cycle mediators [6].

In addition to genetic mutations, epigenetic changes such as hypoxia, muscle tension, or chromosomal rearrangements are key in the propagation of large fibroids via enhanced expression of several genes, including *HMGA2* [7]. Hypomethylation and deregulation of this genetic material trigger the proliferation of the extracellular matrix, which constitutes the bulk of LM [7]. 

Lymphadenopathy in the presence of gynecologic complaints is typically associated with malignancy and pelvic inflammatory disease [8]. Rare cases of benign metastasizing leiomyoma (BML) have been reported in the literature [9]. However, this is more frequently associated with distant metastases to lung or musculoskeletal tissues than lymphadenopathy [9]. Previous research has described the role of inflammatory cells in mediating leiomyoma formation. Of note, the role of inflammatory mediators on the progression or formation of uterine or cervical leiomyomas in post-menopausal women has not yet been clearly established in the existing literature. This case report details a post-menopausal woman with chronic abdominal lymphadenopathy who was found to have multiple benign uterine and cervical leiomyomas found secondary to the evaluation of menorrhagia.

## Case presentation

A 60-year-old G3P3003 female not taking hormone replacement therapy was referred to the gynecology clinic for iron deficiency anemia suspected to be secondary to postmenopausal uterine bleeding. She entered menopause at age 50 and had not had any uterine bleeding since that time. The patient reported severe bleeding with associated fatigue, presyncope, and anemia requiring blood transfusion. The patient reported this condition had been present for at least six months, over which time her systemic symptoms of fatigue and presyncope had become increasingly severe. At the time of presentation, she had not previously sought treatment. The patient had a past medical history of chronic lymphadenopathy, hypertension, and thyroid disease status post thyroidectomy and an additional past surgical history of tubal ligation. The patient reported that she was previously informed of enlarged abdominal lymph nodes found incidentally on pelvic imaging. To her recollection, there was no intervention or follow-up of that finding. Medications include levothyroxine and losartan-hydrochlorothiazide tablets.

Pelvic examination revealed an enlarged uterus consistent with a 20-weeks size. Fibroids and irregular contours were also noted. There were no adnexal masses palpated. No axillary, subclavian or inguinal lymph nodes were palpable on examination. Transvaginal ultrasound relieved numerous masses consistent with fibroids; the endometrial stripe was not well visualized for measurement. Measurements of four fibroids were recorded with the largest being 7.26 cm by 5.80 cm. An endometrial biopsy was attempted; however, pathology showed no endometrial tissue was obtained. The patient was agreeable to definitive surgical management and provided consent for abdominal hysterectomy (TAH) with bilateral salpingo-oophorectomy (BSO).

**Figure 1:**
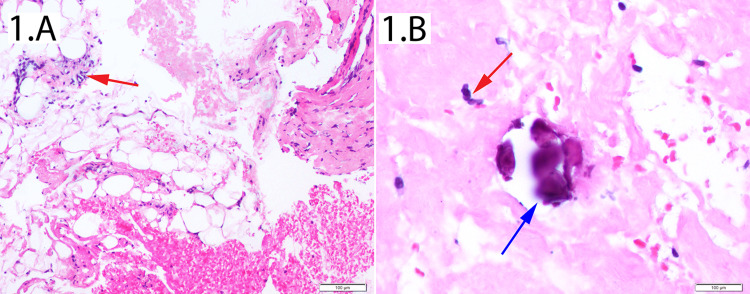
Histopathology of abdominal lymph node biopsy (1.A) This slide shows a global view of a section taken from the CT-guided lymph node biopsy. The red arrow is pointing to plasma cells which are basophilic staining with eccentric nuclei. There is adjacent adipose tissue and a background of amorphous eosinophilic material at the lower right corner of the image. (1.B) This sample is also taken from the lymph node biopsy. The red arrow is again pointing to plasma cells as the blue arrow identifies a focal calcification.

Preoperative abdominal and pelvic imaging revealed new retroperitoneal lymphadenopathy in addition to the existing abdominal lymphadenopathy. These lymph nodes were not palpable on examination. The patient underwent a CT-guided core needle biopsy of a solitary retroperitoneal lymph node (Figure 1). Initial histopathology was consistent with a benign lymph node containing chronic inflammatory cells with micro-calcifications. A background amorphous eosinophilic material suggested amyloid deposition. Specimens were sent to an outside lab for specialized testing. There was no evidence of poorly differentiated cells or ectopic cell lineages. 

**Figure 2:**
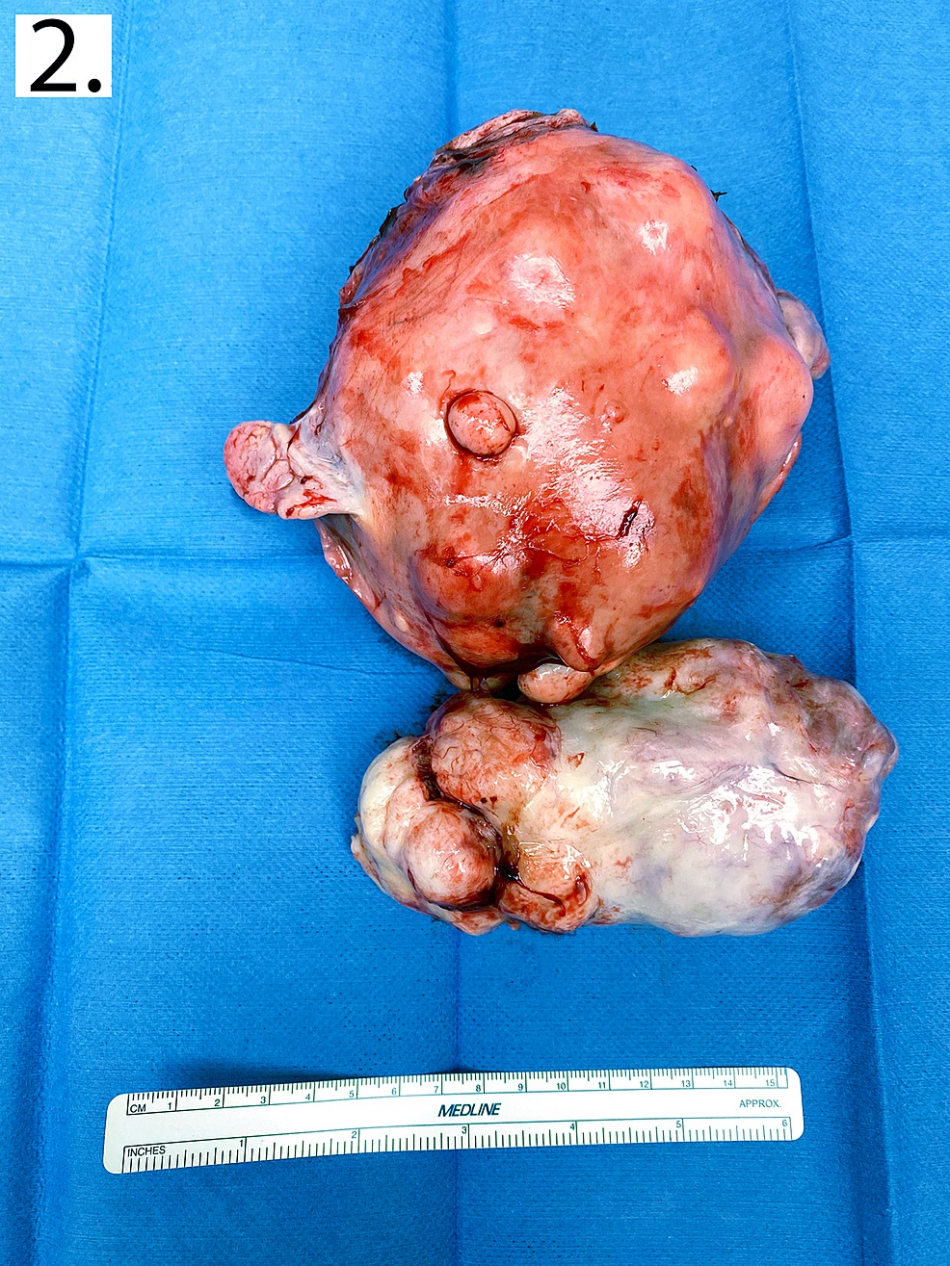
Gross specimen of the uterus and cervical fibroid The two fragments of the uterus had a combined weight of 934 g. The portion of the fundus was 13 by 11 by 9 cm and was distorted by numerous nodules. The second (inferior) fragment is 12 by 7.5 by 6 cm and consists of several large, calcified nodules. The uterine cervix is present but is very distorted by a large cervical leiomyoma. On sectioning, the smaller piece appears to be a single large nodule composed of firm tan tissue. Many of the nodules are calcified which precludes cutting into the specimen.

The patient was taken to the operating room for TAH and BSO by a general obstetrician-gynecologist (OB-GYN). Given the benign appearance of the lymph node specimen, the patient did not provide consent for lymph node dissection. The pelvic cavity was accessed with a midline incision from the pubic symphysis to the umbilicus. Initial inspection of the pelvis showed an enlarged, hyper-vascular uterus with numerous hard submucosal masses (Figure 2). The ovaries were atrophic as expected, and fallopian tubes were surgically absent. A sizable cervical fibroid was encountered. During the vaginal incision, this mass was found to be protruding into the vaginal canal, without mucosal invasion at the time of surgery. The vaginal portion of the mass was sharply dissected from the uterine portion of the mass, and the vaginal mass was clamped and displaced from the vaginal lumen. Following removal of the specimen, no lymph nodes were easily palpable. Following surgery, the patient was taken to the recovery room in stable condition following transfusion of one unit of red blood cells. 

**Figure 3:**
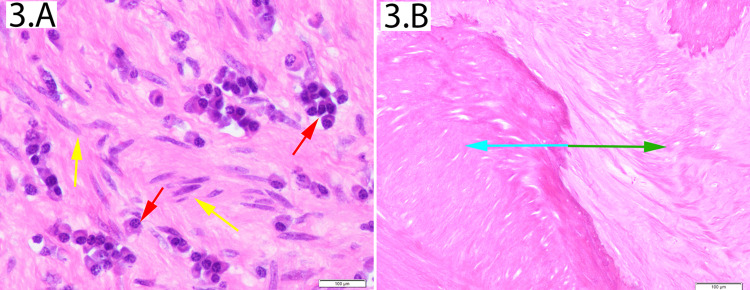
Uterine histology (3.A) This slide is made from a deep sample of a firm uterine tumor. There are bland cigar-shaped smooth muscle cells in a cartwheeling pattern (yellow arrows), consistent with leiomyoma. Plasma cells (red arrows) are present with perinuclear halos. (3.B) This figure shows a sharply demarcated border between the leiomyoma (blue arrow) and normal uterine smooth muscle (green arrow). There is no nuclear staining, indicating necrosis.

Histologic examination of the cervix showed a benign tumor (Figure 3). Plasma cells were infiltrating the leiomyoma, indicating an inflammatory reaction. These findings were like the lymph node findings, indicating a relationship between the two disease processes.

Two weeks postoperatively, the results of the amyloid confirmatory tests returned. The lymph node specimens were evaluated with Congo red Staining to confirm the diagnosis of amyloid deposits. However, the results were inconclusive due to the limited tissue quantity. Light chain testing revealed a polyclonal B cell population with no atypia or abnormal mitoses. 

Upon follow-up several days later, the patient returned to the clinic for removal of skin staples and review of pathological findings. One month post-operation the patient’s symptoms had resolved. She was hemodynamically stable and had not required additional blood transfusions. 

## Discussion

The differential diagnosis of abnormal uterine bleeding (AUB) is broad, comprising structural, and non-structural etiologies. Most causes of AUB in postmenopausal women are in part due to benign changes to the vaginal mucosa and endometrium [10,11]. However, 90% of endometrial carcinomas or neoplasias present as vaginal bleeding, and signs of AUB in any postmenopausal person should be evaluated thoroughly and timely using transvaginal ultrasound and endometrial biopsy [10,12]. Other common structural causes to consider in the differential include polyps (most commonly endometrial), adenomyosis, leiomyoma, malignancy, or hyperplasia [11,13]. Nonstructural causes include endometrial dysfunction, and coagulopathy [13]. Coagulopathy in this group may be primary or secondary to anticoagulant and antiplatelet medications. While ovulatory dysfunction is associated with abnormal uterine bleeding, this is more likely during perimenopause. 

The patient of inquiry in this report underwent appropriate initial management of her presenting symptoms of postmenopausal bleeding, fatigue, anemia requiring transfusion in the setting of a past medical history of chronic lymphadenopathy and hypothyroidism. The ultrasound identified multiple masses throughout the endometrium and cervix. While leiomyomas are the most common benign tumors of the female genital tract, these tend to regress in menopause as the driving ovarian hormones estrogen and progesterone begin to downregulate [14]. However, the literature shows that overt hypothyroidism is significantly associated with LM development even in postmenopausal patients [15]. A history of hypothyroidism in this patient did increase the clinical suspicion of LM prior to tissue biopsy. 

It is important to note the preoperative differentiation of benign LM from malignant uterine sarcoma is not reliable and care must be taken to ensure a diagnosis of malignancy is not missed especially if there is lymph node involvement [16]. Although the presence of post-menopausal uterine fibroids is rare and has a high risk of malignancy, there have been several cases in the literature of benign post-menopausal leiomyomas [9,17].

The chronic lymphadenopathy in our patient was consistent with polyclonal B cell proliferation, not neoplastic tissue or amyloidosis. However, approximately 100 cases of benign metastasizing leiomyoma with diffuse metastasis to the soft tissue, skeletal muscle, pulmonary, and breast tissue have been identified in the literature [9,17]. These lesions are proposed to have hematologic spread in response to prior surgical alteration of the uterus, such as dilation and curettage, myomectomy, or hysterectomy [17]. A viable hypothesis is that cells from the LM could have secondarily spread from a distant location to the lymphatics [17]. However, there was no CT evidence of pulmonary or extrauterine nodules, and the pathologic findings of lymphatic tissue were inconsistent with uterine smooth muscle. 

Instead, in the literature, polyclonal B cell lymphadenopathy is most consistent with a benign disease called persistent polyclonal B-cell lymphocytosis, an indolent stable disease characterized by the presence of abundant binucleated lymphocytes in the marginal zone of the lymph node [18]. Defects in early cell signaling components such as in the nuclear factor kappa-light-chain-enhancer of activated B cells (NF-kB) have been implicated as a potential mechanism for this polyclonal proliferation [19]. These alterations in lymphocyte replication may be unrelated to the epigenetic modifications involved in the pathogenesis of LM as the correlation between these two phenomena have yet to be established. Nevertheless, the presence of both conditions in our patient indicates a point of further research to investigate potential correlation, epigenetic changes involved, and what can be done to prevent these mutations from occurring. 

## Conclusions

A post-menopausal woman with a history of chronic lymphadenopathy, hypothyroidism, and no current or prior use of hormonal replacement therapy, presented for evaluation of severe postmenopausal bleeding leading to iron-deficiency anemia requiring blood transfusion. Further investigation identified multiple large leiomyomas throughout her uterus and cervix. Several enlarged lymph nodes were also identified and sampled for pathology. Since the preoperative probability of malignancy was low due to definitive lymph node diagnosis, this patient was a suitable candidate for total abdominal hysterectomy rather than radical hysterectomy. The patient subsequently underwent a total abdominal hysterectomy, and histologic evaluation revealed no endometrial carcinoma. All tumors identified were benign leiomyomas, and lymph nodes revealed polyclonal B cell expansion. We propose that a similar mechanism involved in the setting of non-hormonal proliferation of uterine smooth muscle through epigenetic modification may relate to the pathogenesis of her concomitant hematological disorder. Although cancer always remains in the differential diagnosis of postmenopausal bleeding, careful consideration of alternative diagnoses makes it possible to select less invasive interventions for low-risk patients.
